# *Ex vivo* model of pathological calcification of human aortic valve

**DOI:** 10.3389/fcvm.2024.1411398

**Published:** 2024-08-29

**Authors:** O. S. Kachanova, N. V. Boyarskaya, P. M. Docshin, T. S. Scherbinin, V. G. Zubkova, V. L. Saprankov, V. E. Uspensky, L. B. Mitrofanova, A. B. Malashicheva

**Affiliations:** Research Laboratory of Diseases with Excessive Calcification, Almazov National Medical Research Centre, Saint Petersburg, Russia

**Keywords:** calcifying aortic stenosis, *ex vivo*, human, pathological calcification, osteogenic differentiation, whole leaflet

## Abstract

The development of drug therapy for the pathological calcification of the aortic valve is still an open issue due to the lack of effective treatment strategies. Currently, the only option for treating this condition is surgical correction and symptom management. The search for models to study the safety and efficacy of anti-calcifying drugs requires them to not only be as close as possible to *in vivo* conditions, but also to be flexible with regard to the molecular studies that can be applied to them. The *ex vivo* model has several advantages, including the ability to study the effect of a drug on human cells while preserving the original structure of the valve. This allows for a better understanding of how different cell types interact within the valve, including non-dividing cells. The aim of this study was to develop a reproducible *ex vivo* calcification model based on valves from patients with calcific aortic stenosis. We aimed to induce spontaneous calcification in valve tissue fragments under osteogenic conditions, and to demonstrate the possibility of significantly suppressing it using a calcification inhibitor. To validate the model, we tested a Notch inhibitor Crenigacestat (LY3039478), which has been previously shown to have an anti-calcifying effect on interstitial cell of the aortic valve. We demonstrate here an approach to testing calcification inhibitors using an *ex vivo* model of cultured human aortic valve tissue fragments. Thus, we propose that *ex vivo* models may warrant further investigation for their utility in studying aortic valve disease and performing pre-clinical assessment of drug efficacy.

## 1 Introduction

Calcifying aortic stenosis is a long-lasting and often asymptomatic condition that manifests itself at the stage of decompensation, when the risk of sudden death increases (1). At the moment, the most effective treatment for this condition is aortic valve replacement. Drug therapy serves as a supportive measure to manage symptoms and prevent complications (2, 3).

The presence of effective strategies to slow down calcium accumulation could potentially lead to the abandonment of surgical treatment for asymptomatic patients with mild to moderate stenosis, provided that there is no negative progression in the degree of calcification and myocardial remodeling (4). In severe stenosis and with hemodynamic disorders, surgery with symptomatic therapy is still the main treatment option. In these cases, anticalcific therapy can be used as a bridge to surgery. Changes in calcification processes after exposure to potential therapeutic targets are being investigated, including lipid and carbohydrate metabolism, the renin-angiotensin-aldosterone system, cyclic guanosine monophosphate signaling, the Notch signaling pathway, and calcium and phosphorus metabolism (4).

Calcifying aortic stenosis is a pathological process characterized by the accumulation of calcium in the aortic valve leaflets, by chronic pressure overload of the left ventricle and by heart failure (5). It has long been believed that the pathogenesis of this condition is based on the gradual mechanical wear and tear of the leaflets over time. However, current research suggests that the development of this pathology may be due to specific molecular processes, which in turn are the result of various factors, including genetics, pressure in the aortic bulb, shear stress, dyslipidemia (abnormal levels of lipids in the blood), inflammation, fibrosis (scarring), and disorders of calcium and phosphorus metabolism (6, 7). Due to the complex and multifaceted nature of the disease, the precise mechanisms underlying aortic valve calcification remain to be fully understood.

Depending on the goals of the researchers, the study of the pathogenesis of aortic stenosis and preclinical testing of anticalcifying drugs (as well as new prosthetics strategies) is carried out using various models. Within the context of research into calcification processes, a diverse range of models can be categorized based on the level of biological organization at which they generate data: molecular, cellular, tissue, system, and organismal. The study of calcification process at the macroorganism level is carried out using biomodels: sheep, since they have the property of spontaneous calcification of valve prostheses (8, 9); pigs (10); alpha-Gal knockout baboons (10). The choice of biological models for calcifying aortic stenosis is limited due to the lack of spontaneous calcification in many of these models. For example, the widely used mouse model does not have a three-layered structure of aortic valves. There are several layers of cells without clearly defined tissue architectonics. Spontaneous calcification does not occur in wild-type mice, but the use of transgenic models solves this problem. Until recently, models knocked out by the genes of the low-density lipoprotein receptor (Ldlr −/−) and apolipoprotein E (ApoE −/−) were widely used (11). It is possible to achieve severe calcification of the aortic valve using this model, however, they did not develop hemodynamically significant stenosis. It can be achieved using the Reversa model, a complex model of hypercholesterolemia with knocked–out Ldlr −/−/apoB 100/100 genes, as well as by introducing a spring guide into the left ventricle that mechanically irritates the valve leaflets, leading to calcification and stenosis (11). An additional factor contributing to hyperlipidemia may be a high-fat diet (12). There are also non-hyperlipidemic calcification models that include MGP modified mice (13, 14), EGFR (11), RBPJk (11), IL1RN (15), but they also lack the development of stenosis. The aortic valves of rats also do not have three-layered structure. The calcification model in rats based on intravenous injections of warfarin does not cause stenosis, and the resulting calcinates differ from those formed in humans (11). Currently, models based on the excessive intake of vitamin D and nicotine are used to induce vascular calcification (16), as well as models involving subtotal nephrectomy and a hyperphosphatemic diet (17). The heart valves of rabbits have a three-layered structure that is similar to those of humans in terms of lipid metabolism. A diet with high cholesterol, in combination with high levels of calcium induced by an excess of vitamin D2, is effective in inducing calcification and stenosis (11). However, the use of the nutrition factor is limited due to the high risk of animal death due to hepatic dysfunction caused by lipid overload (11). Pig valves also have a three-layer construction, and, in contrast to the models mentioned above, pigs are susceptible to the spontaneous development of valve calcium deposits, which can also be exacerbated by the consumption of a high-fat diet (18). Sheep are frequently utilized as models for research on biological prosthetics and homografts *in vivo* (19, 20).

*In vitro* cell cultures are used to reproduce calcification processes at the cellular level. This group of models includes primary cell cultures [interstitial valve cells (VICs) (21), endothelial valve cells (VECs)] (22) and induced pluripotent stem cells (iPSCs) (23). *In vitro* cell culture models are used for both fundamental research and selecting compounds that will be tested later. The advantages of this approach are the relatively short duration of the study and its cost-effectiveness. However, the disadvantage of this model is the need to provide suitable conditions for cell culture (21). In the context of studying calcification of the aortic valve, a structure consisting mainly of an extracellular matrix, this model is not enough to understand the features of interaction between cell populations (including non–dividing ones) and the microenvironment. This is partially compensated by co-cultivation with other cell cultures (5, 24) and the use of 3D cultures and cell culture pump systems (10, 11, 25, 26).

Currently, there is an increasing interest in models that aim to reduce the number of laboratory animals used in research. The “3R” principle—reduction, refinement and replacement—emphasizes the importance of using biomodels more efficiently in experiments (27). There is also a growing demand for bioinformatics models, big data analysis, and machine learning technologies (10, 28, 29). The use of human biological materials in preclinical research is common. These materials are often in the form of cell cultures derived from postoperative specimens and biopsy samples (30–32). The *ex vivo* aortic valve calcification model, which involves the use of native human valve leaflets, seems to be closer to the conditions in the whole organism because of preserving interactions between cell populations and the extracellular matrix. However, the use of this model requires collaboration with medical institutions that are capable of providing biological material. Additionally, appropriate ethical approvals must be obtained, as well as informed and voluntary patient consent. *Ex vivo* models using porcine aortic valves are described in the literature, including evaluating the effectiveness of calcification inhibitors (11, 33–35). Due to the potential for using native valves while preserving the fibrous annulus and the integrity of leaflets, it is now possible to investigate the role of hemodynamic factors in the pathogenesis of aortic stenosis using advanced equipment. Currently, such models are developed using porcine valves (36, 37).

The aim of this study was to develop a reproducible *ex vivo* calcification model based on valves from patients with calcific aortic stenosis. We aimed to induce spontaneous calcification in valve tissue fragments under osteogenic conditions, and to demonstrate the possibility of significantly suppressing it using a calcification inhibitor. To validate the model, we tested the Notch inhibitor Crenigacestat (LY3039478), which has been previously shown to have an anti-calcifying effect on vascular smooth muscle cells (30). We demonstrate here an approach to testing calcification inhibitors using an *ex vivo* model of cultured human aortic valve tissue fragments.

## 2 Materials and methods

### 2.1 Cultivation of valve leaflets

The aortic valve leaflets were provided by the V. A. Almazov National Medical Research Center. The study was conducted in accordance with the principles of the Helsinki Declaration and approved by the Ethics Committee of the V.A. Almazov National Medical Research Center (Ethical authorization No. 12.26/2014). Aortic valve leaflets were obtained from patients undergoing surgical aortic valve replacement procedures after receiving the permission. This study included 14 patients (7 men and 7 women), whose ages ranged from 41 to 76 years (62.79 ± 9.18). 11 patients had severe aortic stenosis, 4 patients had grade 3 aortic insufficiency with mild (*n* = 3) and moderate (*n* = 1) calcification of aortic valve. The peak gradient on the aortic valve in patients with aortic stenosis was 94.9 ± 32.86 mmHg, the average gradient was 54.59 ± 19.5 mmHg, the aortic transvalvular peak velocity was 4.43 ± 1.12 m/s. The final diastolic volume was 162.42 ± 88.43 ml, the final systolic volume was 86.5 ± 71.81 ml, and the Simpson ejection fraction was 52.17% ± 12.48%.

**Figure 1:**
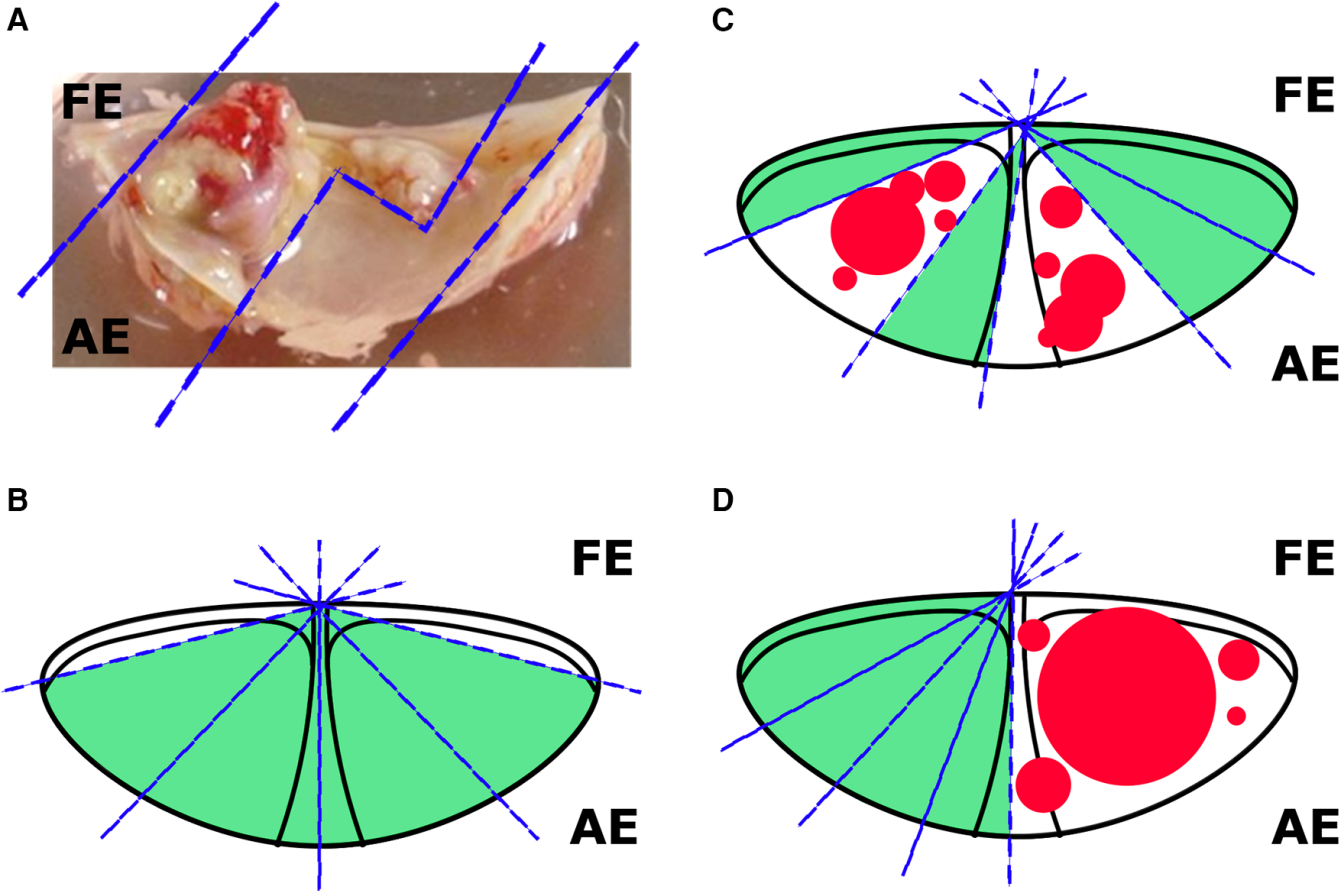
Options for cutting valves for cultivation. **(A)** The sections of a calcified valve available for cutting; **(B)** an example of cutting a slightly calcified leaflet (without visible calcium overlays); **(C)** an example of cutting a moderately calcified leaflet (less than 1/2 of the area); **(D)** an example of cutting a strongly calcified leaflet (1/2 of the area). FE, free edge; AE, anchored edge.

On the day of reception of the entire leaflet (one leaflet from one patient), regions free of calcified plaque were excised for further analysis. The material obtained from a single valve was divided into a number of fragments, which corresponded to the number of experimental groups. The aim of this stage is to achieve maximum uniformity among the fragments and prevent the formation of elevated “calcium background” levels. In order to achieve this goal, we propose visual and microscopic detection of calcium deposits, as well as normalization of fragment sizes to area within a single leaflet. The ways to reduce the “calcium background” by cutting of the calcium deposits is schematically shown in the Figure 1. The cutting was performed within the uncalcified tissues, which was evaluated visually and microscopically. All manipulations were performed under aseptic conditions using sterile equipment. After that, the valve fragments were randomized by experimental groups and placed on 24-well plates (Corning, USA). For further cultivation, the antimyofibroblastic medium was adapted due of the risk of myofibroblast contraction, which can disrupt the penetration of nutrients and the procalcification stimulus (33). A low glucose DMEM medium (Gibco, USA) was used with the addition of 2% fetal bovine serum (FBS, Gibco, USA), 1% insulin-transferrin-selenium (Gibco, USA) and 50 µg/ml gentamicin. The medium was changed twice a week. The fragments were incubated in conditions with a content of 5% CO2 at a temperature of 37 °C. The absence of mycoplasma contamination was verified by quantitative polymerase chain reaction (qPCR) in accordance with Janetzko et al. (38). All experiments were conducted in biological triplicates (cells isolated from at least three donors). Leaflet fragments were cultured for 8 weeks to assess the degree of calcification, as well as for histological assessment. For quantitative polymerase chain reaction (qPCR), the leaflet fragments were cultured for 4 weeks.

### 2.2 Osteogenic medium

Osteogenic medium includes 50 µM of ascorbic acid, 100 nM of dexamethasone and 10 mM of β-glycerophosphate to the culture medium (Sigma, USA). Other cultivation conditions were the same as for the described ones above; the osteogenic medium was also changed twice a week.

Calcium accumulation was assessed by spectrophotometric method using a kit “Calcium Olvex” (Olvex Diagnosticum, Russia) at the 8th week of cultivation in accordance with the manufacturer's protocol with some changes. The fragments of the leaflets were washed twice with PBS, after that they were homogenized in the distilled water (500 µl per fragment). Homogenization was performed mechanically using 2 stainless steel grinding beads to grind one fragment placed in a 2 ml thick-bottomed centrifuge tube. The oscillation process lasted for 30 min and the homogeneity of the product was checked every 15 min. Next, the homogenate was centrifuged at a speed of 300G for 5 min in order to remove the beads and destroyed tissues. The supernatant was moved to the new centrifuge tube and was re-centrifuged at a speed of 1000G for 5 min. 60 µl of the supernatant was placed in centrifuge tube with 500 µl of the Monoreagent from the Calcium Olvex kit, after which, according to the manufacturer's protocol, the mixture was incubated for 5 min at room temperature. After that, the optical density of experimental, calibration and blank samples was measured at a wavelength of 650 (640–660) nm in a cuvette with an optical path length of 1.0 cm on the multi-mode plate reader CLARIOstar® Plus. To measure the area of the valve fragment (S), photographs of each fragment were utilized, which were taken in order to monitor the dynamic accumulation of calcium (photographs for each fragment were taken). Using the ImageJ program, the area of the fragments on all photos was measured (4 photos for each fragment) and the arithmetic mean among them was calculated {thus, for each fragment had one area value [S(average)]}. Then, using the formula C(normalized)=C(non-normalized)/S(average), was obtained a normalized calcium concentration normalized by the area of a particular fragment, that is, the concentration of calcium in mmol/L/cm^2^. Data processing was carried out using Microsoft Excel and GraphPad Prism.

### 2.3 Calcification inhibitors

The gamma secretase inhibitor Crenigacestat (LY3039478; Medchemexpress, USA) was dissolved in DMSO (dimethyl sulfoxide) to a concentration of 10 mM. Dosages were used in studies that showed the minimum number of side effects in patients in clinical trials: 100 nM (Cmax = 46.444 ng/ml), 300 nM (Cmax = 139.332 ng/ml), 500 nM (Cmax = 232.22 ng/ml) (39, 40).

### 2.4 Morphological assessment

On day 1, 2, 4, 8 weeks, the valves were photographed at a magnification of 30x. The morphology of the leaflets and the qualitative assessment of calcification in dynamics were evaluated.

### 2.5 Quantitative polymerase chain reaction in real time

To isolate RNA, the extraRNA reagent (Eurogen, Russia) was used in accordance with the manufacturer's instructions with some modifications. After placing the fragments of the leaflets in the extraRNA reagent, its homogenization was carried out in 2 ml thick-bottomed centrifuge tubes using stainless steel beads (2 beads per fragment). The oscillation lasted no longer than 10 min, and the homogenization quality was checked every 5 min. Next, the homogenate was centrifuged at a speed of 1000G for 5 min, after which the supernatant was placed in new centrifuge tubes. Next, we followed the manufacturer's protocol. After isolation 1 μg of total RNA, was used for reverse transcription using MMLV reverse transcriptase (Eurogen, Russia).

Real-time PCR was performed using SybrGreen qPCR mastermix “qPCRmix-HS SYBR” (Eurogene, Russia) in the LightCycler 96 system (Roshe, Switzerland) according to the following scheme: (1) denaturation before amplification for 300c at 95 °C; (2) 45 cycles of three-step amplification (15c at 95 °C, 30c at 60 °C, 30c at 70 °C); (3) High resolution melting. Expression of GAPDH, RUNX2, COL1A1, SNAIL, SLUG, ACTA2 was evaluated after 4 weeks of osteogenic conditions. mRNA levels were normalized to GAPDH mRNA. All primer sequences are available on request.

### 2.6 Statistics

The data obtained by spectrophotometric calcification assessment and qPCR were processed using Microsoft Excel (calculations) and GraphPad Prism (graphs, statistical analysis). The optical density levels were calculated minus the blank sample. The data was analyzed using ANOVA in GraphPad Prism. The Shapiro-Wilk test was used to check the normality. Since not all samples passed the normality test, the statistical significance of the differences between the samples was assessed using the Mann-Whitney test. Those samples that passed the normality test were tested using the *t*-test. The standard errors of the mean value (SEM) are indicated.

Changes in the expression levels of target genes were calculated using the comparative ΔΔCT method. GAPDH was used as a housekeeping gene. The results are presented as an average of technical duplicates. The standard errors of the mean value (SEM) are indicated. Relative expression levels were compared using the Mann-Whitney test in GraphPad Prism.

### 2.7 Histological assessment

Sections of the aortic valve (1–2 µm) were dewaxed, rehydrated in a series of ethanol and water solutions and stained with hematoxylin-eosin. The morphology of the sections was evaluated using a microscope Zeiss Axio Observer Z1 in the program ToupView.

## 3 Results

### 3.1 Assessment of osteogenic differentiation under *ex vivo* conditions

**Figure 2:**
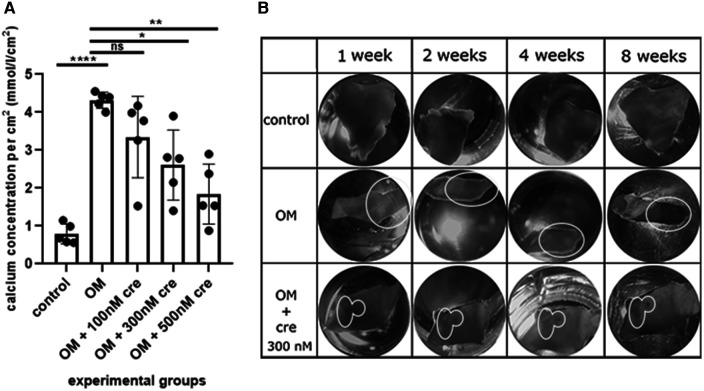
Assessment of calcium accumulation under *ex vivo* conditions **(A)** spectrophotometric assessment of valves in the control, osteogenic medium (OM) and osteogenic medium with the addition of 100 nM, 300 nM, 500 nM crenigacestat (LY3039478). **(B)** Morphological assessment of calcium accumulation in the control, in an osteogenic medium (OM), an osteogenic medium with the addition of 300 nM crenigacestat (OM + cre). The groups were compared using the *t*-test, *p = 0.0111; **p = 0.0022, ****p = <0.0001. The groups were compared using *t*-test.

First, we set up the experimental conditions to culture aortic valve fragments (see Materials and methods section and Figure 1 for details). Then we induced osteogenic differentiation of the valve fragments by culturing them in the osteogenic conditions (Figure 2). After 8 weeks we estimated calcium accumulation by spectrophotometric assessment of valves in control and osteogenic medium and detected a significant accumulation of calcium in osteogenic conditions (Figure 2). During the morphological assessment of the valves (Figure 2B), a distinguishable formation of calcium plaques was found in samples under osteogenic conditions.

### 3.2 Inhibition of calcification by crenigacestat was obtained under *ex vivo* conditions

**Figure 3:**
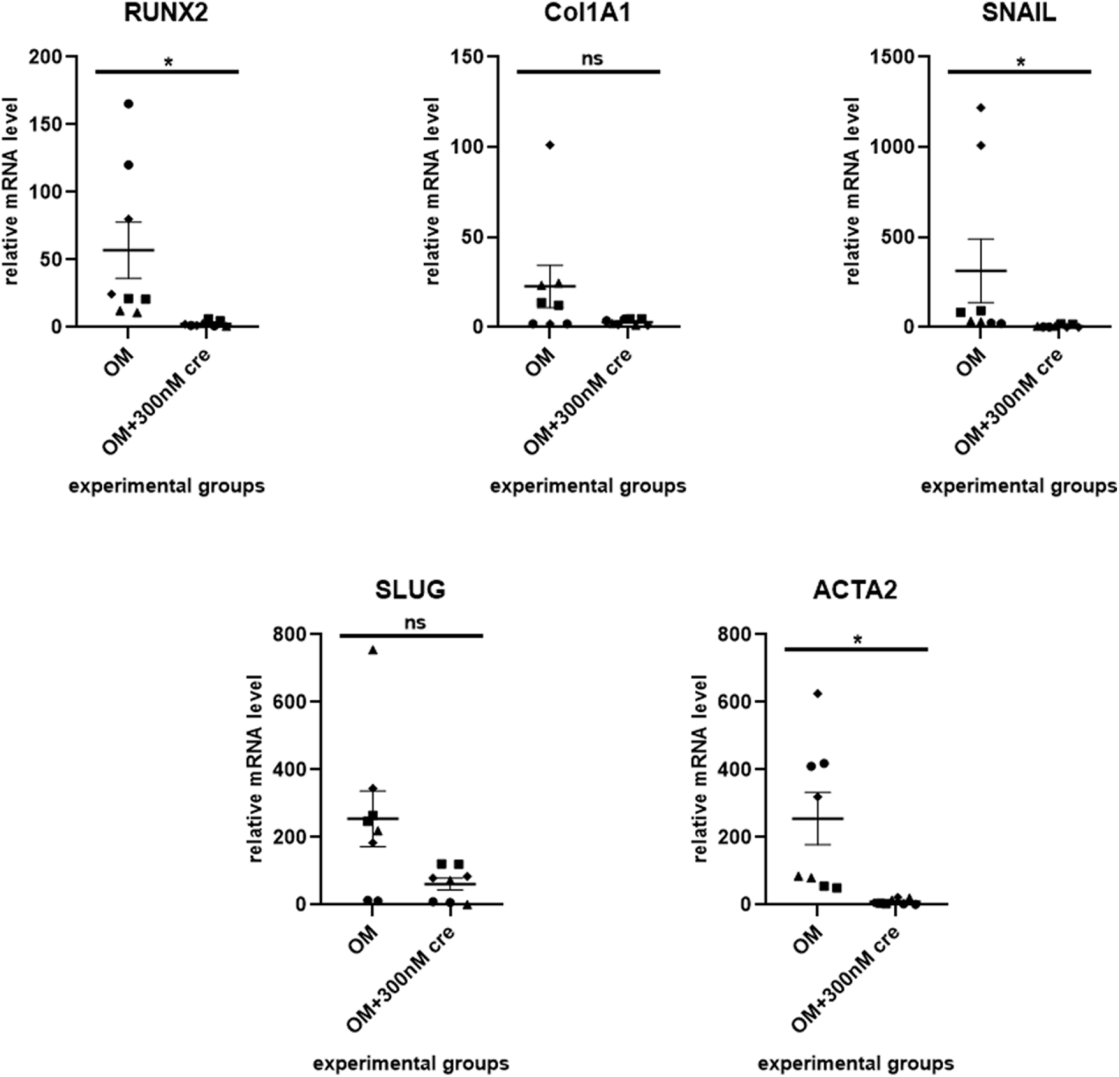
Crenigacestat suppresses the expression of the genes associated with osteogenic differentiation. The effect of crenigacestat (LY3039478) on *RUNX2* (the main regulatory gene for osteogenic differentiation), *COL1A1* (a gene encoding fibrillar protein of the extracellular matrix), *ACTA2* (a gene encoding a protein from the actin family used as a marker of myofibroblast formation) and on markers of endothelial-mesenchymal transition (*SNAIL*, *SLUG*). OM is an osteogenic medium; OM + 300 nM is an osteogenic medium with the addition of 300 nM of crenigacestat. The groups were compared using the Mann-Whitney test, *p < 0.05.

To evaluate the ability of our *ex vivo* culture method for valve fragments to detect the anti-calcification effect of a drug, we used crenigacestat, a substance that has been previously shown to inhibit the osteogenic differentiation of human aortic valve interstitial cells. We cultured valve fragments in either a control medium or an osteogenic medium, or an osteogenic medium supplemented with crenigacestat. A significant suppression of calcium accumulation was found in samples with 300 nM and 500 nM of crenigacestat (Figure 2A). Crenigacestat suppressed the expression of the genes associated with osteogenic differentiation. The effect of crenigacestat (LY3039478) on *RUNX2* (41) (the main regulatory gene for osteogenic differentiation), *COL1A1* (a gene encoding fibrillar protein of the extracellular matrix), *ACTA2* (a gene encoding a protein from the actin family used as a marker of myofibroblast formation) and on markers of endothelial-mesenchymal transition (*SNAIL*, *SLUG*) (Figure 3) (42, 43).

### 3.3 The formation of new foci of calcification in osteogenic conditions was histologically determined

**Figure 4:**
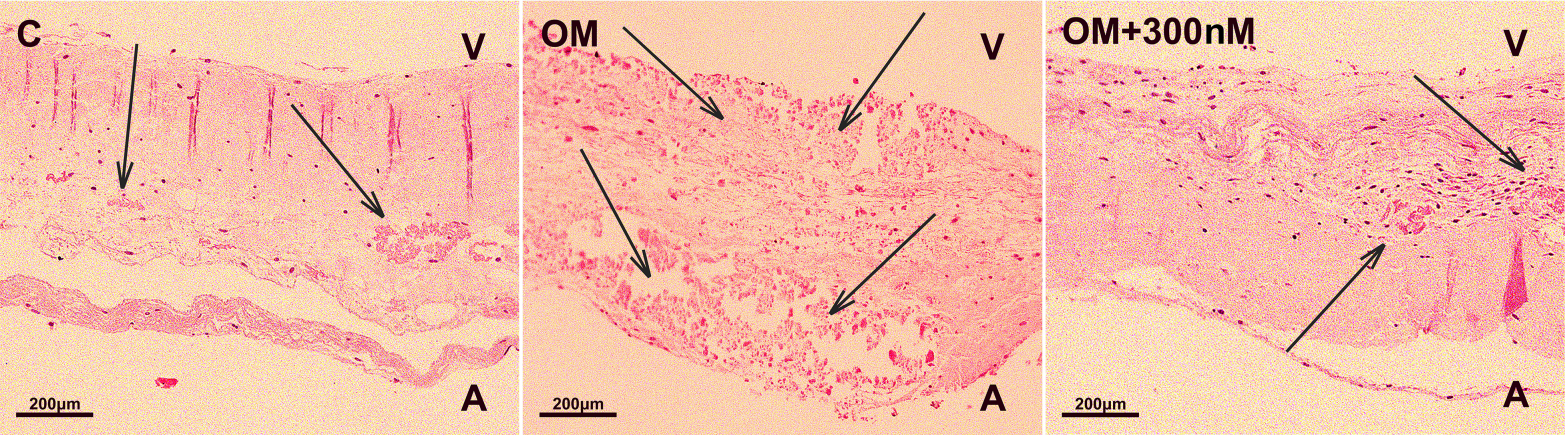
Staining of fragments of the aortic valve leaflets with hematoxylin-eosin of the control group **(C)**, under osteogenic conditions (OM), under osteogenic conditions with the addition of 300 nM crenigacestat (LY3039478). The formation of new foci of calcification in the group with osteogenic conditions (OM) is observed.

Finally, we assessed the valve fragments by histological staining with hematoxilin-eosin to show calcification in osteogenic medium and its suppression by crenigacestat (Figure 4). Newly formed foci of calcification were identified in all samples under osteogenic conditions without the addition of crenigacestat. In the group in osteogenic medium with the addition of 300 nM crenigacestat new calcification foci were observed in the pars spongiosa. In the control group without osteogenic conditions, no newly formed foci of calcification were observed. Microscopically, we found old foci of calcification formed *in vivo* as well as foci of fibrosis, hyalinosis, and cartilaginous metaplasia in the samples, without a clear dependence on the experimental conditions.

## 4 Discussion

The aim of this study was to develop an *ex vivo* calcification model using fragments of aortic valve tissue in laboratory conditions. The fragments were exposed to specific experimental procedures for a in order to induce calcification. The main advantages of this model include the preservation of the histological structure of the valve leaflets and the ability to maintain them in osteogenic conditions for an extended period. As seen from the presented data the model allows to evaluate the expression of genes responsible for calcification and biological activity of an inhibitor in the valve.

Addition of 300 nM crenigacestat into an osteogenic medium led to a significant decrease of *RUNX2* expression in valve tissue fragments compared to the control samples in osteogenic medium. The expression of other genes that are also associated with osteogenic differentiation, such as *COL1A*, *ACTA2*, and *SNAIL*, decreased when the valve fragments that had been osteogenically induced were treated with crengiacestat. All this demonstrates the potential of this *ex vivo* model for testing substances for their ability to prevent calcification.

A major limitation of this study is that it uses *ex vivo* models that use tissues obtained from patients with aortic valve disease. It is difficult to obtain non-diseased tissues with living cells. Difficulties in interpreting the results may be caused by “calcium background” associated with plaques formed *in vivo*.

We have concluded that addressing this issue depends on selecting patients to participate in the study. Leaflets with more than 50% of the valve area affected by calcification were excluded due to technical difficulties in isolating unaffected areas from macroscopically visible calcifications. Additionally, we used microscopy at a 30× magnification to evaluate visible calcinates that were undetectable to the unaided eye. If technically possible, the calcinates were removed. If not, a fragment of the leaflet was left under dynamic observation. Special attention was paid to minimizing the “calcium background” during the process of assessing calcium accumulation using spectrophotometry. For statistical analysis, it was necessary to compare the degree of calcium accumulation within a single leaflet, due to the individual characteristics of patients, which created a different calcium background despite the measures taken. At the same time, if the previously mentioned measures were insufficient, it can be assumed that we would not have observed statistically significant differences, even with such an analysis design. Another way to interpret the results, even in the presence of significant calcium background, is through the use of non-invasive techniques that allow for assessing the degree of calcification over time. We used microscopy with a 30× magnification, but this technique is not sensitive enough to fully detect dynamic changes. Therefore, for these purposes, more sensitive imaging techniques, such as micro-CT, should be employed (11, 44). The use of these techniques would also allow for the implementation of software programs for calculating the area of calcification, thereby providing not only qualitative but also quantitative data (45).

Therefore, we propose that *ex vivo* models of aortic valves derived from patients with calcific aortic stenosis may be worth further investigation in order to determine their potential utility in pre-clinical evaluation of drug efficacy. In addition, this model may be useful for future basic research. Since the preservation of the extracellular matrix and cell composition in the samples would allow for the use of histological methods to detect local differences in the expression of specific markers. However, one of the most important factors of aggression leading to aortic stenosis, mechanical stimulation, is still not observed in this study. It is possible to utilize hemodynamic systems that create circulation of a culture medium. However, to create systems that replicate *in vivo* hemodynamic conditions (i.e., a combination of linear and turbulent flow patterns relative to the ventricular and aortic sides), at minimum, an entire valve with its fibrous ring preserved is required. Such work is carried out with valves of large animals (36). Additionally, a significant limitation of the model is the absence of reliable data regarding whether leukocytes, which are known to play a significant role in remodeling processes, can survive under *ex vivo* conditions. Further investigation is required to address this issue.

## 5 Conclusion

We have developed an *ex vivo* calcification model using aortic valves from patients with calcified aortic stenosis. This model allowed us to demonstrate the calcification-inhibiting effects of the gamma secretase inhibitor crenigacestat (LY3039478). Therefore, we propose that this model warrants further investigation in order to assess its potential utility for studying calcification processes and preclinical trials.

## Data Availability

The original contributions presented in the study are included in the article/Supplementary Material, further inquiries can be directed to the corresponding author.
